# Infliximab-Induced Vanishing Bile Duct Syndrome

**DOI:** 10.7759/cureus.21940

**Published:** 2022-02-05

**Authors:** Michael J Eiswerth, Matthew A Heckroth, Ali Ismail, Dibson D Gondim, Ryan Kaufman

**Affiliations:** 1 Department of Internal Medicine, University of Louisville Hospital, Louisville, USA; 2 Department of Pathology, University of Louisville Hospital, Louisville, USA

**Keywords:** infliximab, elevated liver transaminases, drug induced liver injury, vanishing bile duct syndrome, cholestatic liver injury

## Abstract

Drug-induced liver injury (DILI) is a spectrum of pathology that can be classified by mechanism of injury or by type of observed hepatotoxicity. Vanishing bile duct syndrome (VBDS) is a group of acquired and genetic disorders that cause the destruction and disappearance of intrahepatic bile ducts, and cholestasis. VBDS typically presents with severe cholestatic hepatitis and can have immunoallergic features. Infliximab has been reported to rarely cause a cholestatic pattern of liver injury due to ductopenia characteristic of VBDS. Herein we present a clinical case of infliximab-induced DILI resulting in VBDS.

## Introduction

Drug-induced liver injury (DILI) is a broad term encompassing a variety of mechanisms of hepatic injury. Hepatotoxicity can be induced by drugs and may be either dose-dependent or idiosyncratic [1]. Furthermore, idiosyncratic DILI can be further organized into allergic and non-allergic mechanisms. DILI can also be classified based on the type of hepatic injury as either hepatocellular, cholestatic, or a mixed pattern in addition to pathologic findings, severity, and chronicity [2]. Drug-induced bile duct injury is a type of idiosyncratic injury characterized by severe damage to the biliary epithelium and can be accompanied by the disruption of the biliary tree architecture [3]. Within the biliary system, cholangiocytes are the predominant cell. They promote bile formation and regulation, but have also been shown to be susceptible to immune-mediated injury [4,5]

Vanishing bile duct syndrome (VBDS) refers to a group of disorders in which drugs or other insults lead to destruction or loss of intrahepatic bile ducts due to prolonged cholestatic injury. The underlying pathophysiology occurs when a drug or other insult leads to an inflammatory response directed at cholangiocytes. The resulting bile duct degeneration leads to chronic cholestasis [4,6,7]. VBDS is a pathological diagnosis but can be made clinically in practice. In this condition, "vanishing" refers to the histopathologic loss of intrahepatic bile ducts, with no corresponding radiographic findings. Many cases arise within a few months of onset of severe cholestatic hepatitis and can present with immunoallergic features such as rash, fevers, facial edema, lymphadenopathy, or eosinophilia [8]. The severity and course of VBDS can vary but has previously been reported to present with Steven-Johnson’s syndrome [9] and can even lead to liver failure requiring transplant.

This article was previously presented virtually as a meeting abstract at the 2020 ACG annual scientific meeting which took place from 10/23/21 - 10/28/21.

## Case presentation

A 51-year-old male with stage III melanoma with lymphadenopathy was treated with an immunotherapy regimen of talimogene laherparepvec (TVEC) and pembrolizumab, a programmed death 1 (PD-1) checkpoint inhibitor, for a total of five cycles. The patient’s clinical course was complicated by pembrolizumab-induced colitis. Three weeks after the last dose of pembrolizumab the patient began to experience abdominal pain, malaise, low-grade fevers, and loose bloody stools with mucous. An initial colonoscopy was consistent with autoimmune proctitis for which infliximab therapy was initiated. He received two doses of infliximab until over a two-week period, he experienced progressive watery and bloody diarrhea greater than four times a day with associated low-grade fevers, malaise, and darkening of his urine. Six weeks after initiation of infliximab his liver enzymes were markedly elevated. Prednisone was started at 1mg/kg and infliximab was held. Ultimately, he presented to the hospital with complaints of fever, cough, and malaise and was admitted for further evaluation.

His vitals on admission were within normal limits. Physical exam revealed jaundiced skin and right upper quadrant abdominal tenderness. Labs demonstrated mixed pattern cholestasis with aspartate aminotransferase (AST) of 1408, alanine aminotransferase (ALT) of 627, alkaline phosphate (ALP) of 579, total bilirubin (TBIL) of 16, direct bilirubin of 9.4, with an international normalized ratio (INR) of 1.0 (Table 1). Of note, all of these liver enzymes were within normal limits on recent bloodwork two months prior to admission. High-dose steroids were transitioned to methylprednisolone on admission with ursodiol, N-acetylcysteine, and supportive care. Initial imaging consisted of an abdominal ultrasound showing biliary sludge without gallstones and no acute findings of the liver or biliary tree. A magnetic resonance cholangiopancreatography (MRCP) showed no evidence of biliary obstruction or extra-hepatic ductal disease. An initial liver biopsy on admission was consistent with a drug-induced liver injury or autoimmune hepatitis.

**Table 1:** Evaluation of immunologic and infectious etiologies of elevated liver enzymes. CMV: Cytomegalovirus HSV: Herpes Simplex Virus EBV: Epstein-Barr Virus WBC: White Blood Cells

Parameters	Results	Reference Range (units)
Ammonia	47	9-55 (uMol/L)
WBC	5.4	4.1-10.8 (10^3^ cells/µL)
Mitochondria M2 Antibody	≤20	≤20
Immunoglobulin G	413	750-1,560 (mg/dL)
Immunoglobulin A	<40	80-450 (mg/dL)
Immunoglobulin M	163	46-304 (mg/dL)
Hepatitis A IgM	Nonreactive	(normal, low, nonreactive)
Hepatitis B surface antigen	Nonreactive	(normal, low, nonreactive)
Hepatitis B core antibody	Nonreactive	(normal, low, nonreactive)
Hepatitis B surface antibody	Nonreactive	≥12
Hepatitis C antibody	Nonreactive	(normal, low, nonreactive)
Hepatitis A IgG	Reactive	(normal, low, nonreactive)
Hepatitis E antibodies	Negative	(NA)
QuantiFERON Tuberculosis	Negative (0.14)	<8.0 International Units/mL
EBV DNA, blood	negative	(normal, low, nonreactive)
HSV-1 PCR, blood	Positive	(normal, low, nonreactive)
HSV-2 PCR, blood	Negative	(normal, low, nonreactive)
CMV DNA PCR	Positive	(normal, low, nonreactive)
CMV DNA PCR, Quantitative	195	International Units/mL
Blood cultures	No growth at five days	(NA)
Urine Cultures	No growth	(NA)

Laboratory workup for immunologic and infectious causes of liver enzyme elevation was performed (Table 1). Flexible sigmoidoscopy on admission was significant for diffuse, moderately erythematous mucosa with a loss of vascular pattern and exudate at the anal verge extending 35cm, suggesting autoimmune proctitis similar to the initial colonoscopy. Flexible sigmoidoscopy also was suggestive of cytomegalovirus (CMV) colitis by immunostaining biopsies from the rectosigmoid colon. Ganciclovir was started empirically. An initial liver biopsy was negative for CMV by immunohistochemistry. Per infectious disease recommendation, ganciclovir was transitioned to valacyclovir and a six-week course was completed.

**Figure 1:**
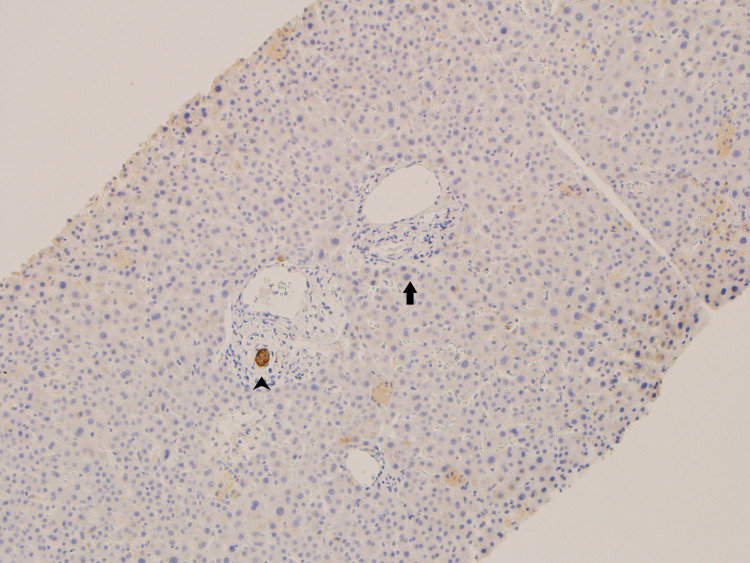
Cytokeratin 7 immunohistochemical stain of the portal tract Cytokeratin 7 immunohistochemical stain highlights the absence of bile duct in the portal tract (arrow). The arrowhead points to a normal-appearing bile duct

**Figure 2:**
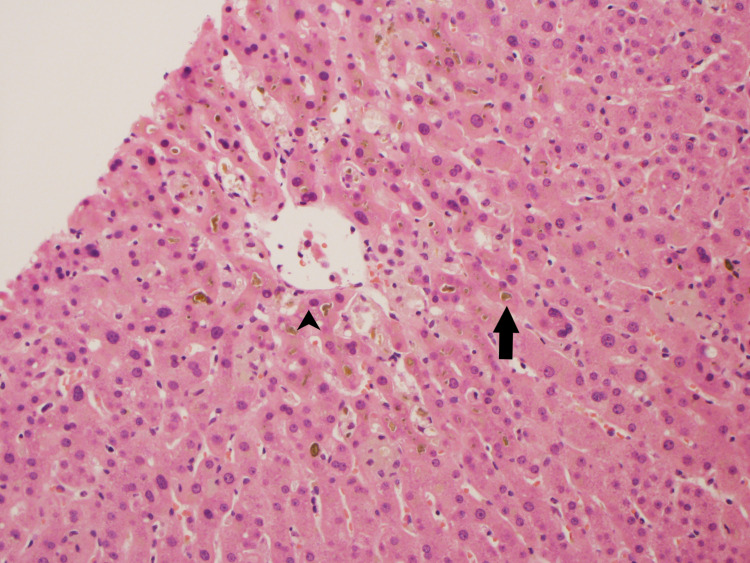
Cholestasis in zone 3 of the liver The arrowhead shows central vein. The arrow shows zone 3 cholestasis.

Over a prolonged hospital course of 30 days, the patient’s liver enzymes slowly trended down although they did remain elevated even at time of discharge (Table 2). Prior to discharge a repeat liver biopsy, performed four weeks after the first liver biopsy, demonstrated extralobular and intralobular degeneration of bile ducts and acute cholestasis consistent with VBDS (Figures 1, 2). No evidence of CMV by immunostaining was noted on repeat liver biopsy. This biopsy was performed 28 days after the initial biopsy, and five months after initial exposure to infliximab. 

**Table 2:** Weekly trend of liver enzyme elevation during hospital course AST: Aspartate Aminotransferase ALT: Alanine Aminotransferase ALP: Alkaline Phosphatase TBIL: Total Bilirubin INR: International Normalized Ratio

Date:	AST	ALT	ALP	TB	INR
1/21/2020	627	1,408	579	16.1	1.0
1/28/2020	228	654	347	14.0	1.3
2/4/2020	142	478	315	17.8	1.1
2/11/2020	123	453	327	15.6	1.1
2/18/2020	191	62	478	17.7	1.0
2/25/2020	171	567	583	23.0	1.0

## Discussion

Infliximab is a monoclonal antibody to human tumor necrosis factor alpha, a drug commonly used in severe inflammatory bowel disease, rheumatoid arthritis, among other diseases. It falls under the classification of disease modifying antirheumatic drug (DMARD). Common side effects include rash and fever but can cause severe hypersensitivity reactions, bone marrow suppression, and reactivation of tuberculosis or hepatitis B. There are at least four forms of hepatic injury that have been associated with infliximab, and this drug has a likelihood score of A of causing liver injury, meaning it is a well-established cause of clinically apparent liver injury [10]. It can induce hepatotoxicity by causing cholestatic liver disease. This cholestasis has been typically described as self-limiting but can manifest with a prolonged clinical course of greater than six months. The clinical courses described in previous literature vary from mild elevation of liver enzymes with improvement over the course of 12 months to severe cases requiring plasmapheresis, and even liver failure [11-13]. In contrast, our patient had previously been on pembrolizumab, which is a frequent cause of cholestatic DILI and can cause elevated liver enzymes in 1-10% of patients [14]. The use of validated scoring systems and review of prior case reports are important to differentiate which medications may be causing DILI. The Adverse Drug Reaction Probability Scale (Naranjo) has previously been used in DILI, and in this patient pembrolizumab was given a score of four, correlating to a “possible” cause [15]. Pembrolizumab has a likelihood score of E and is possibly a cause of clinically apparent liver injury [16]. Given the temporal relationship of infliximab use and onset of DILI six weeks later, it is more likely that infliximab was the driving agent of liver injury, but a close assessment of all medications is important when evaluating for VBDS.

VBDS is primarily a diagnosis of exclusion and although it is not commonly caused by infliximab, it has been recently reported [17]. Like this recent case, our patient had normal liver enzyme tests prior to infliximab use, enzyme levels peaked at six to eight weeks after last use, and ultimately required hospitalization for persistent cholestatic hepatitis. However, it is essential to have a broad differential when evaluating for possible VBDS. Graft-vs-host disease (GVHD), Hodgkin’s disease, primary sclerosing cholangitis (PSC), and primary biliary cholangitis (PBC) are all conditions that can mimic VBDS and must be considered [1]. Viral infections can also cause liver injury leading to VBDS. Our patient had CMV colitis and data from animal studies and previous case reports have demonstrated that CMV infection can be followed by VBDS [18,19]. Our patient had multiple liver biopsies that were negative for CMV, making it less likely in this case.

Diagnosing VBDS is challenging, and as seen with our patient, may require multiple liver biopsies, MRCP, and extensive infectious and immunological workup to rule out alternate pathology. Diagnosis of VBDS does require specific elements: persistent elevation in serum ALP and bilirubin for more than six months after onset of DILI, absence of clinical or serologic evidence for PBC, PSC, or GVHD, and a liver biopsy showing paucity of intralobular bile ducts in a sample obtained at least one month after onset of DILI [1]. VBDS has a spectrum of presentations and is variable in severity with cases ranging from mild disease to life-threatening.

## Conclusions

Infliximab-induced cholestatic hepatitis has been described in previous studies, however the pathophysiology of this condition remains unclear. Further studies into this condition are important and will likely include both genetic and environmental factors. When a prolonged, severe cholestatic pattern of liver injury occurs it is essential to keep VBDS in mind. By definition liver biopsy is required for confirmation of VBDS, however the diagnosis is often made clinically due to its varied spectrum of presentation.
